# Work-Related Challenges among Primary Health Centers Workers during COVID-19 in Saudi Arabia

**DOI:** 10.3390/ijerph18041898

**Published:** 2021-02-16

**Authors:** Khalid Al-Mansour, Abdullatif Alfuzan, Danya Alsarheed, Munwer Alenezi, Fouad Abogazalah

**Affiliations:** 1 Department of Social Studies, College of Arts, King Saud University, Riyadh 11451, Saudi Arabia; Aalfuzan1@ksu.edu.sa; 2 General Administration for Primary Health Centers, Ministry of Health, Riyadh 12628, Saudi Arabia; Dalsarheed@moh.gov.sa (D.A.); Malonezy@moh.gov.sa (M.A.); Fabogazalah@moh.gov.sa (F.A.)

**Keywords:** stress, role conflict, role ambiguity, social support, self-esteem, primary healthcare centers, COVID-19, Saudi Arabia

## Abstract

This study aimed to identify certain occupational risk factors for stress among healthcare workers (HCWs) during the COVID-19 pandemic. Using a multistage random sampling approach, an online questionnaire was applied to collect data on role conflict and ambiguity, self-esteem and social support from 1378 HCWs working in primary health centers (regular and fever clinics; clinics specialized in managing patients with COVID-19 symptoms) across Saudi Arabia. The results showed that stress correlated positively with role conflict and ambiguity and negatively with social support. HCWs in fever clinics exhibited significantly more stress and role conflict and ambiguity than those who were working in regular primary healthcare centers. In conclusion, role conflict and ambiguity and social support were determinants for stress among HCWs, especially those working in fever clinics.

## 1. Introduction

Coronavirus Disease-2019 (COVID-19) is a disease caused by Severe Acute Respiratory Syndrome that was firstly detected in Wuhan, China in December 2019 [1]. By March 2020, the rapid increase of COVID-19 cases in many countries has reached pandemic proportions [2]. On 11 March 2020, the number of confirmed cases was 118,000, with over 4000 deaths [3]. The increase in the number of confirmed cases and deaths continued to reach nearly 50 million confirmed cases and more than 1,245,000 deaths by 8 November 2020 [4].

With regards to Saudi Arabia, the first case was officially reported on 2 March 2020 [5,6,7]. According to the Johns Hopkins Coronavirus Resource Center on November 8, 2020, the number of confirmed cases in Saudi Arabia exceeded 350,000, while the number of deaths was more than 5500 [8]. Therefore, Saudi Arabia has taken many precautionary measures during the pandemic to control the spread of the virus, such as imposing a lockdown, asking people to wear masks in public and launching online education [7,9]. In addition to providing free treatment to all citizens and residents, the country built up healthcare centers, named fever clinics, in all cities and governorates to receive only patients with COVID-19 symptoms [10]. All these efforts could be considered one of the reasons for increasing pressure on the health system, which may lead to a further increase in the level of stress among healthcare workers (HCWs).

Previous national and international studies, however, have shown high levels of stress among HCWs, whether in regular conditions or during the COVID-19 pandemic [6,11,12,13,14,15]. For example, the Middle East Respiratory Syndrome coronavirus (MERS-CoV) has been identified in 2012 in many countries worldwide and most cases were diagnosed in Saudi Arabia [16]. National and international studies by then documented increased stress among HCWs who dealt with MERS-CoV cases, especially those who were working in primary healthcare centers and this stress undermined their quality of healthcare [17,18,19].

In addition to sociodemographic characteristics, several work-related factors were suggested to affect the stress levels of HCWs, including role conflict and ambiguity, self-esteem and social support [20,21,22,23,24]. Role conflict and ambiguity can be attributed to the absence of clear and reliable data about the activities needed in a specific position, the unpredictability of performance results and information deficiency regarding the required tasks [25,26]. Self-esteem can be defined as positive or negative beliefs about oneself and it is also an important personal means to alleviate stress, while social support is seen as an individual’s response to participating in social groups that help support one another [27,28,29]. Psychological stress theory can give us the important of both personal and environmental factors on stress level. Psychological stress theory is a theory that explain the stress and coping process and that theory was developed by Lazarus in his book Psychological Stress and the Coping Process in 1966 [30]. According to Lazarus and Folkman (1986) stress is defined as “a relationship with the environment that the person appraises as significant for his or her well-being and in which the demands tax or exceed available coping resources” [31]. The stress can result from two types of factors and these factors are personal and environmental factors [31]. General beliefs including self-esteem can be one of the personal factors that influence the appraisal among individuals [32]. According to Lambert and Lambert [33], self-esteem is one of the factors that might influence the level of stress among nurses. Different environmental factors might influence stress including constraints and resources [32]. Several studies found constraints such as work conflict and ambiguity can affect the level of stress among health care professionals as constraints [34,35]. Also, Social support as environmental resources can affect the level of stress among healthcare workers [36].

However, no study, to date, has investigated the environmental and personal impacts on the levels of stress among HCWs in Saudi Arabia during the COVID-19 pandemic. We also have no information on whether HCWs in fever clinics specialized to manage patients with COVID-19 symptoms experience the same stress levels and personal and environmental stressors as their counterparts in regular primary healthcare centers in Saudi Arabia. Thus, this research was conducted to identify the association between role conflict and ambiguity, self-esteem and social support with stress levels among HCWs in primary healthcare centers in Saudi Arabia. We also objected to assess the differences in stress, role conflict and ambiguity, self-esteem and social support between HCWs in regular healthcare centers and fever clinics.

## 2. Materials and Methods

This cross-sectional study was conducted on HCWs in the primary healthcare centers in Saudi Arabia using a multistage random sampling technique. Saudi Arabia is divided geographically into five regions; Central, Eastern, Western, Northern and Southern regions. The Saudi Minister of Health is represented in each region by the Directorates of Health Affairs (*n* = 20). Each directorate supervises regular primary healthcare centers (*n* = 2070) and fever clinics (*n* = 119). In this study, we randomly selected six regular health centers and two fever clinics from all directorates and sent emails to all workers in the selected centers and clinics, including electronic questionnaires, before sending reminders one week later. Data collection was started on 27 September 2020 and continued for three weeks. The anticipated time to complete the survey was approximately 10 min. Based on power analysis with G*Power software edition 3.1.9.7 for linear multiple regression analysis (with seven predictors, at least a medium effect of 0.10 and 95% power), the required sample size is between 235 and 245.

### 2.1. Participants

Physicians, nurses, other health professionals (lab specialists, radiologists, pharmacists, social workers and epidemiologists) and other workers (administrators, receptionists and managers) who worked in the primary healthcare centers were the target population. All employees working (medical and non-medical jobs) in each randomly selected center were invited to participate in the study by using the official emails from the Ministry of Health. The total number of invited persons was 2007, of whom 1378 replied, representing a response rate of 69%. The head of the primary healthcare center in each Health Affairs Directorate coordinated the data collection process in each health directorate. The principal author was available to contact when having any questions or facing any difficulties. The head of the primary healthcare center in each directorate of health affairs emailed the self-administrated survey link to the targeted participants. During the time frame of data collection, a weekly reminder was mailed to the targeted participants.

### 2.2. Study Variables

This study had one dependent variable (perceived stress) and several independent variables (role conflict and ambiguity, self-esteem and social support). Other covariates included age, gender, smoking, marital status, nationality, educational level, the type and the site of the healthcare center (regions) and job.

### 2.3. Measures

#### 2.3.1. Stress

The Perceived Stress Scale (PSS) was used to measure stress in this study. The PSS has been vastly utilized as a measure of stress estimate with strong psychometric properties [37]. It includes ten items to appraise an individual’s stress. The participants evaluated the degree to which life stressors were overpowering and unmanageable over the preceding month, on a scale ranging from 0 (never) to 4 (very often) and a higher score refers to a higher level of stress [37]. According to Cohen et al. [38], PSS has good validity since it is highly correlated with different scales such as self-reported health and health services measures, health behavior measures, smoking status and help-seeking behavior. Furthermore, research shows excellent validity and reliability with healthcare professionals in Saudi Arabia [39,40,41]. The scale in the current study has good reliability and validity (Cronbach’s alpha 0.85). According to Chaaya et al., the Arabic version of the Perceived Stress Scale is valid [42].

#### 2.3.2. Self-Esteem

Self-esteem is defined by Rosenberg [43] as a positive or negative attitude toward the self. The current study used the Rosenberg Self-Esteem Scale (RSES), which has been used significantly. The scale consists of 10 items as self-report and assessed participants based on a 4-point Likert scale varying from 1 (strongly disagree) to 4 (strongly agree) [44]. A higher score on the scale means higher self-esteem. Several studies have evaluated the reliability and validity of the RSES and found that the scale has acceptable reliability and validity [45,46]. In this study, Cronbach’s alpha coefficients for this scale equaled 0.72. Also, the scale has a good criterion validity since it has a significant relationship with the Perceived Stress Scale (*r* = 0.142, *p* < 0.01).

#### 2.3.3. Social Support

Social support is explained as “understood or real instrumental and/or meaningful provisions supplied by the group of society, social networks and confiding” [47]. The Multidimensional Scale of Perceived Social Support (MSPSS), which was used in this study, identifies perceptions of support from three vital dimensions of individuals’ emotional support: family, friends and significant others. The MSPSS is composed of 12 self-administered items [48]. Responses choices are in the form of a 7-point Likert scale ranging from 1 (very strongly disagree) to 7 (very strongly agree) with a higher score on the scale reflecting higher social support. The MSPSS is validated for internal consistency and is acceptable for reliability [46,49,50,51]. Our study showed that it had good reliability (Cronbach’s alpha 0.92). In addition, the scale is valid based on criterion validity (*r* = −0.220, *p* < 0.01).

#### 2.3.4. Work Role Conflict

Role conflict is defined as the concurrent event of at least two job pressures so that the consistency with one makes it very hard to respond to the next [52]. Bowling et al. (2017) developed a role conflict measure for workers, which was used in this study to assess work-role conflict among primary healthcare workers. It includes six measured items and the participants can respond to the questions on a 7-point Likert scale ranging from 1 (strongly disagree) to 7 (strongly agree) with a higher score on the scale pointing to a higher level of conflict. There is ample reliability and validity evidence depend on [53]. In the present study, the scale has a reliability (Chronbach’s Alpha = 0.62), as well as criterion validity with the perceived Stress Scale (*r* = 0.472, *p* < 0.01).

#### 2.3.5. Work Role Ambiguity

This variable is defined as “reflecting certainty about duties, authority, allocation of time and relationships with others; the clarity or existence of guides, directives, policies; and the ability to predict sanctions as outcomes of behavior” [54]. The Ambiguity instrument measures work role ambiguity using six items measured on a 7-point Likert scale from 1 (strongly disagree) to 7 (strongly agree). A higher total score for role ambiguity means the respondent experiences more work role ambiguity. The reliability and validity of this scale have been tested everywhere [53]. As shown in the current study, the scale has good reliability (Chronbach’s Alpha = 0.85). Also, the scale has a good criterion validity with perceived Stress Scale (*r* = 0.436, *p* < 0.01).

### 2.4. Demographic Variables

First, age was measured in this study as a continuous variable. Second, gender was coded in this study to be male (1) and female (0). Third, the nationality of the health care workers was coded to be a dichotomous variable as Saudi (1) and non-Saudi (0). Fourth, marital status was a dichotomous variable as married (1) and not married (0). Fifth, education level was coded to be high school or less (0) and a university degree (1). Sixth, smoking in this study was coded as yes (1) and no (0). Seventh, type of job was recoded as medical and health job (1) and non-medical and health job (0). Eight, type of the health center was coded as regular health care center (1) and fever clinic (0). Ninth, regions were dummy coded into four dummy variables as following: Eastern (dummy 1), Western (dummy 2), Northern (dummy 3) and Southern (dummy 4). The Central region was used as a reference variable.

### 2.5. Ethical Considerations

This study was approved by the Central Institutional Review Board of the Ministry of Health in Saudi Arabia. The informed consent was on the first page of the electronic survey and included a description of the study, including the purpose and the names of the researchers. Anonymity was ensured since the names and I.P addresses were not collected. Also, to protect the confidentiality of the participants’ data, the data were saved on the primary researcher’s password-protected personal computer and no one had access to the data except the research team. Participants had to select “agree to participate” and “submit response” for their participation to be considered.

### 2.6. Data Analysis

Data were imported into the statistical software Statistical Package for the Social Sciences (SPSS, version 25) to analyze the study data. This study used a variety of statistical analysis procedures to analyze the data and the level of statistical significance was set to 0.05 for all used statistical tests. First, sociodemographic and occupational data were described. Second, Pearson correlation was used to examine the relationships between stress and all sociodemographic and occupational characteristics in addition to the workplace challenges. Third, linear regression analysis was used to assess the relationship between stress and the statistically significant factors in the correlation using two steps; step 1: control variables were entered and step 2: role conflict, role ambiguity, social support and self-esteem were entered. Fourth, independent sample t-tests were used to examine the differences between HCWs in regular and fever clinics in the levels of stress, role conflict and ambiguity, self-esteem and social support.

## 3. Results

The mean age (standard deviation) of participants was 38.9 (8.12) years; 908 (65.9%) were aged 31 to 45 years old, 771 (56.0%) were males, 1158 (84.0%) were married and 1171 (85.0%) were citizens. Regarding their job, 306 (22.2%) were physicians, 432 (31.3%) were nurses, 131 (9.5%) were other HCWs, 509 (39.9%) were administrative workers, 1101 (79.9%) were working in a regular primary healthcare center and 277 (20.1%) were working in fever clinics (Table 1).

Using Pearson correlation, stress correlated will all outcomes variables; positively with role conflict, role ambiguity and self-esteem and negatively with social support (*r* = 0.472, 0.436, 0.142 and −0.220; respectively, *p*-values <0.01 in all correlations). Stress also correlated with other control variables; positively with a job and Eastern and Western regions (*r* = 0.096, 0.083 and 0.074) and negatively with age, gender and center type (*r* = −0.307, −0.227 and −0.069; respectively) (Table 2). Several demographic variables were not significant, including (marital status, nationality, education level, smoking, Northern and western). These insignificant variables will not be included in the MRA because they violated the assumption of linearity.

In step 1, stress was associated with age (*β* = −0.234, *p* < 0.01), followed by gender (*β* = −0.157, *p* < 0.01), nationality (*β* = 0.099, *p* < 0.01) and center type (*β* = −0.067, *p* < 0.05). These factors explained 13% of stress. In step 2, age (*β* = −0.176, *p* < 0.01) and gender (*β* = −0.149, *p* < 0.01) remained associated with stress. In addition, role conflict (*β* = 0.314, *p* < 0.01), role ambiguity (*β* = 0.193, *p* < 0.01), social support (*β* = −0.099, *p* < 0.01) and self-esteem (*β* = 0.088, *p* < 0.01) were also associated with stress. Eventually, all of these factors together explained 35.4% of perceived stress, therefore, it could be concluded that workplace challenges in the form of role conflict, role ambiguity, social support and self-esteem contributed to 22.4% of stress (Table 3).

HCWs in fever clinics were shown by t-test to have higher levels of stress, role conflict and role ambiguity than their counterparts in the regular healthcare centers. The values of means (standard deviations) for fever clinics versus regular healthcare centers were as follows: stress: 18.70 (7.62) versus 17.33 (7.54), *p*-value= 0.007, role conflict: 23.82 (7.19) versus 22.85 (6.86), *p*-value= 0.038 and role ambiguity: 17.56 (8.74) versus 16.19 (8.32), *p*-value= 0.016, respectively (Table 4).

## 4. Discussion

This study indicated that role conflict and ambiguity, self-esteem and social support were significant predictors for stress among HCWs in Saudi Arabia during the COVID-19 pandemic. Besides, HCWs who were working in fever clinics specialized to manage people with COVID-19 in Saudi Arabia had higher stress levels and role conflict and ambiguity than their counterparts who were working at regular primary healthcare centers.

The increased stress levels among HCWs in fever clinics who were on the frontlines during the COVID-19 pandemic came in line with previous national and international studies assessing stress among similar study populations. In a study conducted on 426 HCWs from Saudi Arabia and Egypt (48.4% physicians, 24.2% nurses and 27.4% other HCWs) on the frontlines fighting COVID-19, more than half (55.9%) of them had stress [55]. Another study conducted on 5062 HCWs (19.8% physicians, 62.2% nurses and 18.0% technicians) treating patients with COVID-19 in China showed that 29.8% of participants had stress [56]. One study conducted on 346 HCWs from Spain (90% of them were general practitioners) found that 63% of HCWs experienced stress during the COVID-19 pandemic [57].

In comparison with the general population or the HCWs managing diseases other than COVID-19, HCWs who inspect and treat patients with COVID-19 are more vulnerable to psychological deficits, including stress, for many reasons [58,59,60]. First, they are more likely to get infected with COVID-19 and consequently transmitting the infection to their family, friends and lovely ones. Second, they suffer a hefty workload that they have to work for more hours and attend extra shifts. Third, the worldwide shortage of personal protective equipment had a stressful impact on the mental status of HCWs. Fourth, in many cases, HCWs had to take meticulous precautions while they examine patients, which adds extra psychological burdens [55,61].

Furthermore, we could find that HCWs in fever clinics had higher levels of role conflict and role ambiguity than other HCWs in regular primary healthcare centers in Saudi Arabia. To the best of our knowledge, this is the first study to assess role conflict and ambiguity among HCWs from Saudi Arabia during the COVID-19 pandemic. The fever clinics were recently initiated in Saudi Arabia as a response measure to tackle the spread of COVID-19 across the country; therefore, it is not unexpected that HCWs in these new clinics do not have enough details on their job descriptions. They also might have incomplete knowledge of infection control precautions. Above all, the changing guidelines regarding COVID-19 precautionary and management protocols can be perplexing for HCWs managing COVID-19 patients. On the other hand, HCWs in other regular primary healthcare centers should have been occupying their places for years, so they are expected to have clear job descriptions, enough knowledge of the diseases they manage and strict protocols regulating their work. This discrepancy in job descriptions and knowledge of diseases could be the reason behind the differences in role conflict and role ambiguity between HCWs in fever clinics and regular primary healthcare centers in Saudi Arabia.

Moreover, our results demonstrated that role conflict and role ambiguity reported by HCWs in fever clinics were associated with their stress levels. This finding agrees with the results of previous studies conducted on workers from various disciplines. A self-report questionnaire administered to 66 health professionals in Italy showed that role ambiguity constituted a psychosocial deficit that influenced workers’ wellbeing and led to emotional exhaustion [62]. In a cross-sectional study conducted on 2989 Japanese employees, work ambiguity was associated with psychological distress and even mediated the relationship between job insecurity and psychological distress [63].

Also, we could detect a negative correlation between social support and stress among the studied HCWs. In a previous study, Saudi HCWs who lacked emotional support from family and society during the COVID-19 pandemic showed higher levels of stress compared with their counterparts who received family and social support [55]. This finding highlights the need for providing social support to HCWs on the frontlines to alleviate their psychological stress.

Unexpectedly, we could notice a positive association between stress and self-esteem, which contradicted previous findings among HCWs [64,65]. While it is difficult to justify this finding, we could speculate that the high self-esteem among HCWs in our study could be partially explained by their experience in managing MERS-CoV pandemics. Thus, HCWs in Saudi Arabia who are stressed due to managing COVID-19 have good self-esteem due to the previous management of MERS-CoV. Still, more research is needed to confirm our findings. Still, the possibility of residual confounding cannot be excluded.

## 5. Strength and Limitations

This study poses many strengths that should be addressed, such as examining for the first time the status of role conflict and ambiguity among HCWs in Saudi Arabia during the COVID-19 and determining their association with stress, investigating a large sample, using a random sampling procedure to guarantee representativeness of the study population and allow extrapolation of results and assessing the exposures and outcomes using validated questionnaires. However, some limitations should be considered. First, the cross-sectional design of this study does not imply causality; therefore, prospective cohort studies are advised to confirm our findings. Second, some confounding variables that might have affected the results were not assessed, such as the availability of personal protective equipment and medical specialty. Third, due to the strict measures of social distancing, we had to access participants via e-mails; therefore, the possibility of non-response bias cannot be excluded since the sociodemographic and occupational characteristics of responders might have differed from non-responders. For example, HCWs with a busy schedule who probably have higher stress levels might have less time to check their e-mails and respond to the questionnaire [66].

## 6. Conclusions

This study revealed higher levels of stress and role conflict and ambiguity among HCWs in fever clinics during the COVID-19 pandemic than HCWs in regular primary healthcare centers in Saudi Arabia. Besides, role conflict and ambiguity, social support and self-esteem were found to be major determinants of stress among HCWs during the COVID-19 pandemics. Future programs and interventions should be considered to minimize role conflict and role ambiguity in fever clinics in Saudi Arabia to deliver the most efficient health services to patients with COVID-19.

## Figures and Tables

**Table 1:** Socio-demographic Characteristics of studied participants.

Variable	No. (1378)	%	Mean	SD
Age			38.9	8.12
Less than 30 years	197	14.3		
31 to 45 years	908	65.9		
46 years or older	273	19.8		
Gender				
Male	771	56.0		
Female	607	44.0		
Marital Status				
Married/Living w/partner	1158	84.0		
Divorced/Separated	54	3.9		
Widowed	11	0.8		
Single	155	11.2		
Nationality				
Citizen	1171	85.0		
Not Citizen	207	15.0		
Education Level				
High school or less	117	8.5		
Two years of college	652	47.3		
Bachelor	500	36.3		
Graduate	109	7.9		
Type of health center				
Regular PHC	1101	79.9		
fever clinic PHC	277	20.1		
Job				
Physician	306	22.2		
Nurse	432	31.3		
Other health professionals	131	9.5		
Other	509	39.9		
Smoking				
Yes	271	19.7		
No	1107	80.3		
Site of health center				
Central	235	23.6		
Eastern	128	9.3		
Western	337	24.4		
Southern	441	32.0		
Northern	147	10.7		

**Table 2:** Pearson correlation [*r*] between stress and other work-related challenges.

Variable	1	2	3	4	5	6	7	8	9	10	11	12	13	14	14	16	17
Stress	1																
Age	−0.307 **	1															
Gender	−0.227 **	0.220 **	1														
Marital status	0.033	−0.026	−0.023	1													
Nationality	−0.047 *	0.201 **	0.268 **	−0.078 **	1												
Education	0.033	−0.026	−0.023	−0.051	−0.456 **	1											
Center Type	−0.069 *	0.002	0.004	−0.011	0.022	−0.024	1										
Job	0.096 **	−0.137 **	−0.279 **	−0.038	−0.305 **	0.163 **	−0.024	1									
Smoking	0.001	−0.019	0.332 **	0.031	0.147 **	−0.076 **	−0.021	−0.166 **	1								
Eastern	0.083 *	−0.029	−0.128 **	−0.026	0.044	0.090 **	−0.190 **	0.078 **	−0.024	1							
Southern	0.074 *	−0.028	−0.124 **	0.015	0.061 *	0.017	−0.061 *	0.049	−0.006	−0.077 **	1						
Northern	−0.014	−0.067*	−0.008	−0.037	−0.040	0.029	0.052	0.035	−0.066 *	−0.144 **	−0.115 **	1					
Western	−0.041	0.076 **	0.093 **	0.036	0.013	−0.074 **	0.042	−0.065 *	−0.008	−0.075 **	−0.060 *	−0.112 **	1				
Conflict	0.472 **	−0.173 **	−0.025	−0.054 *	0.201 **	−0.016	−0.056 *	0.033	0.045	0.027	0.060 *	0.030	0.030	1			
Ambiguity	0.436 **	−0.164 **	−0.120 **	−0.091 **	0.128 **	−0.005	−0.064 *	0.101 **	0.011	0.065 *	0.059 *	0.006	0.039	0.508 **	1		
Social Support	−0.220 **	0.029	0.034	0.057 *	−0.104 **	0.042	0.049	−0.029	0.011	−0.013	−0.038	−0.010	−0.012	−0.208 **	−0.260 **	1	
Self-Esteem	0.142 **	−0.049	0.004	0.001	0.026	−0.059 *	−0.037	0.052	0.010	−0.016	−0.021	0.031	0.057 *	0.098 **	0.235 **	−0.321 **	1

* *p* < 0.05 level (2-tailed). ** *p* < 0.01 level (2-tailed).

**Table 3:** Linear regression analysis: work-related predictors of stress.

	Step 1				Step 2			
Variable	*B*	*SE*	*β*	*t*	*B*	*SE*	*β*	*t*
Age	−0.179 **	0.027	−0.234	−6.713	−0.136 **	0.023	−0.176	−5.838
Gender	−1.996 **	0.431	−0.157	−4.628	−1.891 **	0.374	−0.149	−5.059
Nationality	1.686 **	0.613	0.099	2.748	0.183	0.537	0.011	0.340
Job	0.711	0.474	0.053	1.502	0.172	0.409	0.013	0.421
Eastern	0.630	0.723	0.029	0.872	0.390	0.625	0.018	0.624
Southern	0.666	0.810	0.027	0.821	0.511	0.701	0.020	0.729
Center Type	−1.031 *	0.497	−0.067	−2.073	−0.515	0.430	−0.033	−1.197
Conflict					1.730 **	0.177	0.314	9.760
Ambiguity					1.239 **	0.209	0.193	5.928
Social Support					−0.052 **	0.015	−0.099	−3.382
Self-esteem					1.466 **	0.480	0.088	3.053
Constant	29.03 **	1.51		19.24	16.946	2.211		7.665
Adjusted *R^2^*		0.13				0.354		
*F*		19.59 **				44.23 **		

Step 1: control variables (age, gender, nationality, job, regions and type of the healthcare center) and step 2: further dependent variables (role conflict, role ambiguity, social support and self-esteem) * *p* < 0.05 level (2-tailed). ** *p* < 0.01 level (2-tailed).

**Table 4:** T-tests and Descriptive Statistics for Stress, Conflict, Ambiguity, Self-esteem and Social Support by Type of Health Center.

Variables	Regular Center		Fever Clinic		* p*-Value
	Mean	SD	Mean	SD	
Stress	17.33	7.54	18.70	7.62	0.007
Role conflict	22.85	6.86	23.82	7.19	0.038
Role ambiguity	16.19	8.32	17.56	8.74	0.016
Self-esteem	18.17	4.04	18.54	3.92	0.167
Social Support	61.86	12.68	60.30	12.73	0.068

## Data Availability

Data is available upon reasonable request by contacting the corresponding author.
